# A Contrast-Guided Approach for the Enhancement of Low-Lighting Underwater Images

**DOI:** 10.3390/jimaging5100079

**Published:** 2019-10-01

**Authors:** Tunai Porto Marques, Alexandra Branzan Albu, Maia Hoeberechts

**Affiliations:** 1 Department of Electrical & Computer Engineering, University of Victoria, Victoria, BC V8P3E6, Canada; aalbu@uvic.ca; 2 Ocean Networks Canada and Department of Computer Science, University of Victoria, Victoria, BC V8N1V8, Canada; maiah@uvic.ca

**Keywords:** image enhancement, image dehazing, underwater image analysis

## Abstract

Underwater images are often acquired in sub-optimal lighting conditions, in particular at profound depths where the absence of natural light demands the use of artificial lighting. Low-lighting images impose a challenge for both manual and automated analysis, since regions of interest can have low visibility. A new framework capable of significantly enhancing these images is proposed in this article. The framework is based on a novel dehazing mechanism that considers local contrast information in the input images, and offers a solution to three common disadvantages of current single image dehazing methods: oversaturation of radiance, lack of scale-invariance and creation of halos. A novel low-lighting underwater image dataset, *OceanDark*, is introduced to assist in the development and evaluation of the proposed framework. Experimental results and a comparison with other underwater-specific image enhancement methods show that the proposed framework can be used for significantly improving the visibility in low-lighting underwater images of different scales, without creating undesired dehazing artifacts.

## 1. Introduction

Recent developments in the infrastructure employed for the exploration of underwater environments such as remotely operated vehicles (ROVs) and cabled ocean observatories have allowed for long-term monitoring of underwater sites. Ocean Networks Canada (ONC), an initiative of the University of Victoria, installs and maintains cabled ocean observatories that include instruments with sensors continuously measuring physical (e.g., pressure, conductivity, and temperature) and biological (e.g., chlorophyll and oxygen) data, as well as cameras and hydrophones. Many time-series from ONC observatories now extend more than a decade.

Since 2006, ONC has captured more than 90,000 h of underwater video from mobile ROVs and camera systems at fixed locations that contain important information for the understanding of marine environments from the coast to the deep sea. This massive amount of data imposes a difficult challenge for scientific analysis, given that the manual investigation of that volume of imagery would require prohibitive amounts of time. Automated methods to perform and assist marine image annotation—which consists in assigning labels to objects or events observed in the images—have been proposed since the 1950s [1]. In order for marine image annotation software [2] to properly operate, the underwater images must provide a reasonable level of visibility (i.e., good contrast and lighting), otherwise the relevance of their results is severely reduced.

Underwater imagery from seafloor cameras is often acquired in low-lighting conditions, which are caused by the use of artificial light sources yielding a non-uniform spatial distribution of the lighting. Low-lighting images are difficult to analyze, which affects their scientific value. Since the contrast in such images varies greatly with the spatial location, it is hard to consistently detect key spatial features such as edges, corners, and intensity gradients. Low-lighting images captured underwater present additional light attenuation challenges, given that they are subject to inherent properties of the underwater environment: absorption, which changes the light energy while it moves through the water based on its wavelength, and scattering, in which particles in the water reflect and deflect the light of objects on the way to the camera. These phenomena cause blurring as well as the loss of contrast and color. Together, they can have a significant negative impact on the performance of higher-level computer vision tasks such as object localization and classification (identification of marine life, counting individuals, etc.).

These types of applications motivate the present research study, which aims at enhancing the quality of low-lighting underwater images by using a single-image contrast-guided approach.

### 1.1. Background

While simple methods can be used to globally enhance the lighting of an image (e.g., by changing the “value” channel in the HSV color space), they will affect all regions of an image (bright and dark) equally, which usually has undesirable effects on regions not requiring enhancement. Other methods [3] look for specific regions of the image with lower pixel intensities, and then attempt to enhance these particular regions by brightening them. In low-lighting underwater enhancement, not only illumination issues should be addressed, but also absorption and scattering.

Schettini and Corchs [4] distinguished the methods proposed to boost visibility in underwater images in two groups: (i) *image restoration*, where an underwater image formation model such as the Jaffe–McGlamery [5,6] is used; or (ii) *image enhancement*, where criteria are used to increase the perceived quality of the image. Examples of image restoration methods are the work of Hou et al. [7], which added underwater optical properties into traditional image restoration approaches, and that of Trucco et al. [8], where a simplified version of the Jaffe–McGlamery model is used to restore underwater images. Notable image enhancement methods (those that do not rely on physical image formation models) are the ensemble of filters used to correct color, contrast and lighting issues of Bazeille et al. [9]; the underwater color correction method of Chambah et al. [10]; the work of Iqbal et al. [11], where underwater images are enhanced by adjusting their contrast, saturation and intensity; and the histogram equalization framework created specifically for underwater images by Hitam et al. [12].

Multiple underwater image processing methods [13,14,15,16,17,18,19] make use of aerial dehazing techniques, given that the issues that plague hazy images (absorption and scattering) create outputs that are similar to those captured underwater. In fact, there is a strong similarity between the optical models of outdoor hazy scenes (as detailed in Section 2.1) and underwater scenes [16]. These methods focus on adapting the aerial dehazing frameworks so that they can be properly used in underwater scenes. Chiang and Chen [13] estimated the depth and lighting sources of underwater scenes, and compensated for light scattering and color change, resulting in haze-free and color-corrected outputs. Ancuti et al. [16] performed color-preserving dehazing in underwater images using a color reference image and a novel color transfer strategy. Yang et al. [14] estimated and removed the perceived blurriness in underwater images by slightly modifying an aerial dehazing technique [20]. Peng et al. [17] also looked at the blurriness of underwater images, noting that objects placed farther from the camera are blurrier (and, equivalently, hazier in aerial images), and then used aerial dehazing methods to increase their visibility. Berman et al. [19] calculated color attenuation ratios and tried different models of water types to reduce the underwater image enhancing task into a regular single image dehazing. Some underwater-specific methods that do not make use of aerial dehazing techniques include the work of Cho and Kim [21], where the authors derived a degradation model specifically suited for underwater images applied to simultaneous location and mapping (SLAM), and the work of Fu et al. [22], which describes an underwater image enhancing model based on a variation of the Retinex model. Given the successful application of aerial dehazing techniques in underwater image processing, we now turn to some background information on haze and dehazing techniques which inform the novel method presented in this paper.

Haze is a weather condition formed by particles and water droplets located in the atmosphere that both scatter and absorb lighting. The light that would ideally reach the observer is not only attenuated by such particles, but also mixed with the atmospheric light reflected by those same bodies (often referred to as *airlight*) [20,23]. The absorption and scattering are mathematically modeled as a linear combination of the direct light attenuation and the airlight [24]. This phenomenon leads to a loss of contrast and color fidelity, especially for distant objects (in fact, the haze is an important visual cue used by humans to infer scene depth).

Haze removal methods attempt to increase the visibility of the scene and correct the color shift that is inherently present in hazy images. Multiple computer vision algorithms may benefit from the haze-less version of the image [20]: from low-level image analysis techniques that search for edges, up to high-level approaches that try to identify objects and infer depth based on visual information. The main challenge in removing haze, fog or smoke from an aerial image is that real-world depth information for the scene is usually unavailable. Knowledge about depth is necessary to remove haze when using a hazy image model (see Section 2.1). Numerous approaches have been proposed to address this challenge, but most of them were initially limited by the need for multiple reference images as input.

He et al. [20] introduced a single-image dehazing method that became extremely popular because of its effectiveness and simplicity. It initially calculates the *dark channel*, a single-channel image formed by the lowest-intensity pixels inside a moving patch on the hazy image with all color channels considered. Then, it uses an assumption called *Dark Channel Prior* (DCP), which states that “non-sky regions in an image have, inside a patch of given size, at least one pixel with very low intensity in one of the color channels”. This empirical observation facilitates the calculation of transmission maps and ultimately the recovery of the haze-less version of the image. This method received a lot of attention and was used and modified by numerous researchers, and recent comparative results [25,26,27] show that this DCP-based dehazing technique is still one of the best options to choose from in terms of performance and accuracy on the dehazing task when only a single image is available.

Dehazing techniques are also used to enhance low-lighting aerial images. Dong et al. [3] observed that the darker parts of low-lighting images behave similarly to haze in their inverted versions. Therefore, by inverting images and removing their “haze”, one would actually remove the darkness and improve the lighting in the original, dark image. Ancuti et al. [28] boosted the visibility in low-lighting images by proposing a modification of the DCP-based dehazing method. This modification considers a non-uniform distribution of lighting sources and also uses the Laplacian of the original image as inputs. In this paper, we apply this idea, i.e., that low lighting can be treated as inverted haze, to underwater images with poor lighting conditions. Other approaches [29,30,31] use convolutional neural networks (CNN) to extract meaningful features from datasets composed of low-light/normal-light pairs of images, and then offered low-lighting enhancement frameworks. Jian et al. [32] recently proposed a CNN-based enhancement network for low-lighting images that does not require normal-light reference images in the training process.

### 1.2. Contributions

In this article, we deal with the phenomena causing visibility deterioration in underwater images using a modification of an aerial-based dehazing method [20], as done previously in similar scenarios [13,14,16,17,18]. Our case study focuses on underwater images that suffer from poor lighting conditions, a common issue observed in images captured at profound depths. To the best of our knowledge, no prior work has employed DCP-based techniques to improve the visibility of low-lighting underwater images using local contrast information as a guiding parameter. Our proposed framework uses an image dehazing pipeline based on the DCP to enhance the visibility of low-lighting underwater scenes using a novel contrast-guided approach for dark channel and transmission map calculation. The method solves three common problems of single-image dehazing methods: oversaturation of radiance, halos around regions with strong gradients, and sensitivity to scale in different-sized images. Differently from Schettini and Corchs [4], we refer to *enhancement* as the process of using an image formation model to recover a version of the input low-lighting underwater image with better visibility.

A novel low-lighting underwater image dataset called *OceanDark*, which is composed of multiple low-lighting underwater image samples captured by ONC using artificial lighting sources, was created to quantitatively and qualitatively evaluate the proposed framework (see Section 3.1). A preliminary version of this work was published in [33]. The theoretical aspects and results of this initial version are significantly expanded in the present article; namely, through the creation of a novel contrast-guided approach for transmission map and dark channel calculation, and the introduction of the *OceanDark* dataset as a basis of evaluation of our proposed method.

The remainder of this paper is structured as follows. Section 2 summarizes the DCP-based dehazing method of [20], and details a novel dehazing approach that can be used to dehaze both aerial and underwater images under different scenarios. Section 3 describes the *OceanDark* dataset and evaluates the proposed approach by comparing it to the DCP-method [20] and four underwater-specific image enhancement methods [18,19,21,22]. Section 4 summarizes the contributions of this article and discusses potential directions for future work.

## 2. Proposed Approach

**Figure 1:**
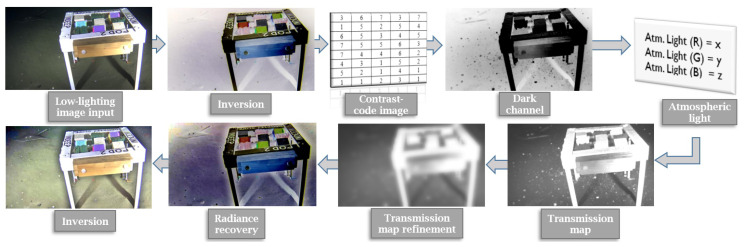
Low-lighting underwater image enhancement framework applied to a sample low-lighting underwater image from *OceanDark*. The image is initially inverted, and then put through a novel, contrast-guided dehazing process before being inverted once again. The output better highlights important features and structures of the original image that were not clearly visible in the original image.

We propose a novel, contrast-guided computational pipeline that solves important issues of state-of-the-art image enhancement methods that use the Dark Channel Prior. Our method is used for the enhancement of low-lighting underwater images. The remainder of this section is structured as follows. Section 2.1 and Section 2.2 discuss necessary background information about the DCP-based dehazing approach proposed in [20], as well as an image filtering operation introduced in [34] and expanded in [35]. These works served as our immediate inspiration and thus we include a detailed discussion of their contributions as a preamble of our original work. Our novel approach is introduced and detailed in Section 2.4. Figure 1 summarizes the image-enhancing framework created.

### 2.1. Dcp-Based Dehazing of Single Images

Note that the discussion in this section involves aerial hazy images, as in the original paper [20]. The formation process of a hazy image can be modeled with Equation (1):

(1) I(x)=J(x)t(x)+A(1−t(x))

where *x* is a vector denoting both spatial coordinates of the image, *I* is the observed hazy image, *J* is the scene radiance (haze-free image), *A* is a scalar representing the global atmospheric light, and *t* is the transmission map, which represents the amount of light that reaches the camera without scattering in the air. Transmission map *t* and haze-less image *J* have the same size, m×n, as the input image *I*. For hazy *color* images, the haze-less image *J* and the vector *A* are composed by three color channels, however *t* is still a single-channel image. Given the model of the hazy image formation in Equation (1), the goal of dehazing is to find *J*, the haze-less version of the image, by inferring the values of *A* and *t*.

The first step in this process is to calculate the *dark channel* (DC). The dark channel is a single-channel image that is populated by the pixel with the lowest intensity in a patch Ω of given size, considering all three R, G, and B color channels of the hazy image. For an arbitrary image *J*, its dark channel $J^{dark}$ can be calculated as:

(2) $$J^{dark}\left(x\right)=\min_{y\in \Omega (x)}\left(\min_{c\in (r,g,b)}J^{c}\left(y\right)\right)$$

where *x* and *y* represent, respectively, spatial coordinates in the haze-less image and inside patch Ω. The Dark Channel Prior is based on the empirical observation that in non-sky regions, for a given pixel location, at least one of its R, G or B values is significantly low. The work in [20] validated this by analyzing 5000 non-sky images and observing that 75% of their dark channel’s pixels were zero-valued, while in 90% of the cases they have intensities lower than 25 (where pixels valued at 255 represent the white color, and 0-valued pixels represent black). Therefore, the dark channel of a haze-less image is going to be mostly black. Note that the size of Ω plays an important role in the amount of dark (i.e., intensities close to zero) pixels in the dark channel.

The second step in the dehazing pipeline of He et al. [20] uses the dark channel to infer the atmospheric light *A* (see Equation (1)). Multiple approaches were proposed to estimate the value of three-dimensional vector $A^{c}$ (where c∈{R,G,B} represents the intensity for each color channel) using single hazy images: looking at the highest intensity pixels in the hazy image [36], calculating the entropy of a percentage of the brightest pixels in the dark channel [37], etc. In [16,20], the 0.1% highest-intensity pixels of the dark channel are first selected, and then the highest-intensity occurrence out of the pixels in the same positions in the hazy image is selected as value for *A*. Recent works [28] have also considered multiple lighting sources in low-lighting settings. After testing different choices of parameters, our approach considers the 0.2% brightest pixels in the dark channel, and then the mean value of their counterparts in the hazy image (similarly to Xiao and Gan [37]). The intensity value chosen as reference in the hazy image provides the three composing values of $A^{c}$.

Once the atmospheric light *A* is obtained, and considering the The Dark Channel Prior, He et al. [20] derived a simplified equation for the calculation of transmission map t(x):

(3) $$t\left(x\right)=1-\omega \min_{y\in \Omega (x)}\left(\min_{c}\frac{I^{c}\left(y\right)}{A^{c}}\right)$$

The transmission map t(x) is a single-channel image of same size m×n as the input hazy image that represents, at each pixel location, the amount of light from the original scene (without the airlight) that reaches the observer. In Equation (3), $A^{c}$ is the atmospheric light in each color channel for images with pixel intensities valued in the range [0,255]. Therefore, after normalizing the hazy image $I^{c}\left(y\right)$, the resulting image pixels will be valued within [0,1]. A phenomenon called *aerial perspective* states that the haze formed by particles in the air is a useful visual cue for distance perception. Therefore, the constant ω(0≤ω≤1) is introduced to preserve some haze and create more realistic images. If ω=1, all haze will be removed in further steps, while ω=0 would preserve the totality of the detected haze. Appropriate values of ω will vary based on each application.

The scene radiance (haze-less version of the image) can be recovered using the calculated atmospheric light *A* and transmission map t(x):

(4) $$J\left(x\right)=\frac{I(x)-A}{\max\left(t\left(x\right),t_{0}\right)}+A$$

The recovered scene radiance (haze-less image) is prone to noise in haze-dense regions, where the denominator in Equation (4) is close to zero. A parameter $t_{0}$ is thus introduced to limit the lower value of this denominator; this parameter preserves some haze, in a similar manner to constant ω in Equation (3).

**Figure 2:**
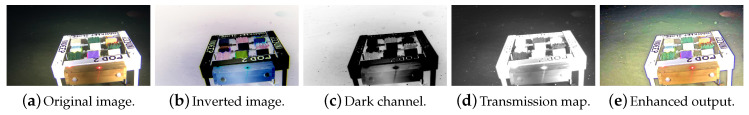
*OceanDark*’s sample enhancement process using static patch sizes of 5×5 and ω=0.9. The original image (**a**) is first inverted, turning the dark regions into hazy segments (**b**). The dehazing process involves the calculation of the dark channel (**c**) and transmission map (**d**) of the inverted image. The haze-less version of the image is obtained and inverted again for the final output (**e**).

The focus of our proposed approach is the enhancement of low-lighting underwater images. The pipeline consists in inverting the image, dehazing it, and then inverting the result again. The physical phenomena responsible for causing haze and fog in land-based images are different from those that cause visibility issues in underwater images. In the latter, turbidity and wavelength-dependant light propagation (nonlinear attenuation of light), among others factors, influence the visibility and color of the scene. Underwater image formation models such as the McGlamery model [6] describe how an underwater image is formed and how it can be enhanced by the calculation of two parameters: a transmission component and a back-scatter light (similar to the atmospheric light *A* of Equation (1)). As noted by Ancuti et al. [16], the model of McGlamery [6] fails to capture the non-homogeneous attenuation of colors characteristic to underwater scenes (refer to Section 3.3 for a discussion on the consequences of this). However, in terms of image composition, the two scenarios (aerial and underwater) can be interpreted in a roughly similar way, which is why the same image enhancement framework can be used for both. Figure 2 illustrates the use of the original dehazing process of He et al. [20] for low-lighting underwater image enhancement.

The *OceanDark* sample of Figure 2a is first inverted (all pixel intensities *x* are changed to $\bar{x}=255-x$) so the darkness in the low-lighting image then represents haze in its modified version. The original dehazing process described in this su-section is applied to remove such haze, yielding the enhanced image of Figure 2e. The enhancement significantly reduces the dark regions of the original image, highlighting structures that were barely visible, such as the fish in the top-right corner of the output. However, the dehazing process used in the example of Figure 2 uses fixed-sized patches of 5×5 and unfiltered transmission maps, which can create halos around regions with strong intensity gradients and false textures.

These dehazing artifacts can be mitigated by a process called transmission map refinement or by varying the size of the patch Ω used in the dark channel and transmission map calculations. Our proposed framework is built considering a thorough analysis on how to execute these two steps: in [33], we determined that the *fast guided filter* is an appropriate image filter to be used, and, in the present work, a novel systematic approach to select the best size for patch Ω is introduced (see Section 2.4 and Section 3.2.2).

### 2.2. Transmission Map Refinement

Recovering the scene radiance using the raw (unfiltered) transmission maps obtained with Equation (3) can lead to the creation of halos in the output images. This happens because the transmission of the image is not consistent throughout a given patch Ω (in particular for larger patches). To mitigate this effect, the transmission map can be refined using an image filter before its use in the radiance recovery phase described by Equation (4). The most commonly used image filters in the related literature are Gaussian filters, bilateral filters [38] and guided/fast guided filters [34,35]. In [33], we use the NYU-Depth V2 [39] dataset, which offers pairs of haze-less images and their depth maps, to synthesize 100 new hazy images and ground truth transmission maps. The hazy images are used to calculate transmission maps, which are then filtered and compared to the ground truth references. The comparative experimental analysis that we reported in [33] concluded that the fast-guided filter is the most suitable for the refinement task.

**Figure 3:**
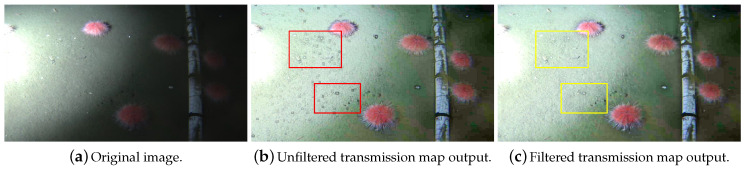
Comparison between enhanced images obtained using unfiltered (**b**) and filtered (**c**) transmission maps. The halos created around regions of significant intensity gradients in (**b**) are greatly diminished with the use of filtered transmission maps in (**c**). The red bounding boxes in (**b**) highlight regions particularly afflicted with halos, whereas the yellow bounding boxes of (**c**) show these same regions with halos of considerably reduced size.

Figure 3 illustrates the negative impact of using raw transmission maps in a dehazed image obtained using ω=0.9 and Ω of 11×11. Figure 3b shows halos around regions of the image where strong gradients (i.e., edges, corners) are present. Little rocks, which represent abrupt color intensity changes in Figure 3a, are surrounded by the undesired halos in the unfiltered transmission map output (Figure 3b). Figure 3c shows the results of dehazing images based on the filtered version of the transmission map. The halos of Figure 3b are replaced by smoother transitions in Figure 3c.

**Fast guided filter**. First introduced by He et al. [34] and then improved in [35], this edge-preserving filter considers the content of a guiding image (which can be a different image or the input image) to execute the filtering task. The filter behaves better than the bilateral filter [38] near edges and can transfer structures from the guiding image to the processed output [34]. This filter generates pixels $q_{i}$ in an output image in terms of pixels $I_{i}$ of a guidance image using Equation (5):

(5) $$q_{i}=a_{k}I_{i}+b_{k},\forall i\in w_{k}$$

Variable *k* refers to the index of a local square window *w* of radius *r*. $a_{k}$ and $b_{k}$ are constant for each $w_{k}$. After minimizing the reconstruction error, He et al. [34] offered Equations (6) and (7) for the calculation of the coefficient maps $a_{k}$ and $b_{k}$:

(6) $$a_{k}=\frac{\frac{1}{\left|w\right|}\sum_{i\in w_{k}}I_{i}p_{i}-\mu_{k}{\bar{p}}_{k}}{\sigma_{k}^{2}+\epsilon }$$

(7) $$b_{k}={\bar{p}}_{k}-a_{k}\mu_{k}$$

Variables $\mu_{k}$ and $\sigma_{k}^{2}$ are, respectively, the mean and variance inside window $w_{k}$ in the guidance image *I*, while |w| indicates the number of pixels inside such window. The input image *p* has a mean pixel intensity of $\bar{p}$ inside $w_{k}$ and parameter ϵ controls the degree of smoothness of the filtered image. The authors of [34] further introduced a method to accelerate the calculation of the filter, called *fast* guided filter [35]. This method consists in sub-sampling both the input and guidance image by a ratio of *s*, and then applying the box filters in the reduced versions of the images, thus obtaining coefficient maps *a* and *b*. These maps are then up-sampled to their original size, now called $\tilde{a}$ and $\tilde{b}$. Finally, the filtered image *q* can be obtained using $\tilde{a}$ and $\tilde{b}$ in Equation (5).

### 2.3. Disadvantages of the Use of Single-Sized Patches

The size of the patch Ω in Equations (2) and (3) used with the minimum operator in the calculation of both the dark channel and the transmission map is a critical parameter for the result and the overall quality of the dehazed output. He et al. [20] shows that using smaller patch sizes such as $\Omega_{3\times 3}$ (i.e., 3×3-sized patches) creates brighter dark channels, making the DCP less accurate. As a result, the recovered scene radiance is oversaturated. On the other hand, larger patch sizes (e.g., $\Omega_{15\times 15}$) create undesired halos around regions with noticeable gradients (see examples in Figure 3b). The survey in [40] further reinforces this concept with the observation that increasing the size of patch Ω creates halos in the recovered images, however using small patches allows pixels of bright objects to be included in the dark channel, yielding inaccurate atmospheric lighting estimations. This trade-off highlights the necessity of customizing the size of patch Ω for each particular application.

The DCP states that pixels of non-sky regions will likely have a low-intensity value in one of their color channels. Increasing the size of patch Ω also increases the likelihood of such low-intensity pixels to be present in the dark channel. However, assigning a single transmission value for increasingly larger patches becomes less representative of the original image and halos become more apparent. Conversely, non-sky regions of small patches might not possess a dark pixel (low-intensity), which contradicts the DCP.

**Figure 4:**
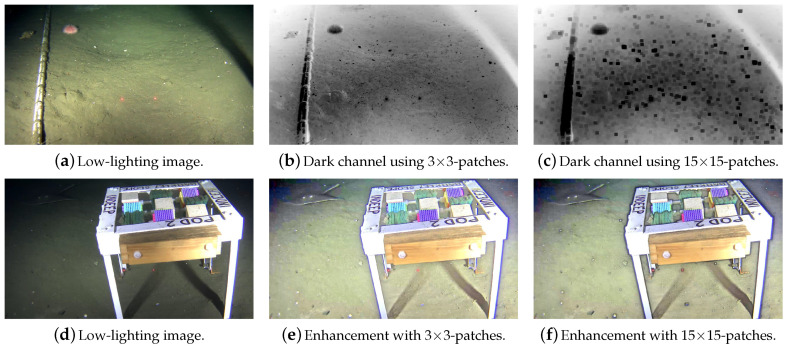
Dehazed scenes and dark channels obtained using $\Omega_{3\times 3}$ and $\Omega_{15\times 15}$. The first row shows a low-lighting image (**a**) and its DC when using $\Omega_{3\times 3}$ (**b**) and $\Omega_{15\times 15}$ (**c**). The mean intensity of the latter is lower, resulting in a darker DC. The second row shows the results of enhancing a low-lighting image (**d**) with $\Omega_{3\times 3}$ (**e**) and $\Omega_{15\times 15}$ (**f**).

Figure 4 shows the effects of different patch sizes in the dark channel calculation and in the overall image enhancement process. A small patch ($\Omega_{3\times 3}$) and big patch ($\Omega_{15\times 15}$) are chosen to illustrate such effects (these are some of the most commonly used patch sizes [40]). The dark channels in Figure 4b,c are calculated using $\Omega_{3\times 3}$ and $\Omega_{15\times 15}$, respectively. The DCP does not reasonably represent the dark channel created using a small patch Ω (Figure 4b), thus the transmission map and recovered image will incorrectly estimate the amount of haze to be removed. The reasoning behind that is the following: hazy regions have higher pixel intensities in the dark channel because their composing pixels have, overall, higher intensities in all three channels of the RGB color space. In fact, the pixels’ intensities in the dark channel are proportional to the haze denseness [20], which represents the amount of light that is attenuated because of this atmospheric phenomenon.

Because of the small 3×3 patch Ω used in the enhancement, Figure 4e has oversaturated pixel intensities, while Figure 4f (enhanced using 15×15-sized patches) recovered colors closer to those of the original image but created halos around strong gradients.

A clear trade-off presents itself in the dehazing task: smaller patch sizes will incorrectly estimate the haze denseness, resulting in the oversaturation of the radiance (i.e., pixel intensities) in the output image. On the other hand, bigger patch sizes create undesired dehazing artifacts that introduce halos. An additional issue is that the same static (i.e., fixed) patch size is not adequate for images of varying sizes. Specifying a single patch size for images with possibly complex and heterogeneous content might lead to a non-ideal fit for their different regions. A method that accounts for two patch sizes (3×3 and 15×15) was proposed [41], but it fails to account for intermediate scenarios where neither option of size is appropriate. Generally, one may encounter two opposite scenarios, which are briefly described below:

1. Hazy regions in the image: The pixel intensities do not drastically change in these regions, because of their more homogeneous color distribution. Therefore, patches of different sizes (i.e., ranging from 3×3 up to 15×15) will likely capture a pixel that is closely-valued to the mean intensity inside the patch, correctly representing the transmission of this sub-region. In this case, bigger patch sizes are preferred given that they will strengthen the DCP by creating darker dark channels.
2. Regions with complex content: In these regions, selecting a big patch size will oversimplify the dark channel and transmission map, creating halos in the dehazed image. Since there are multiple intensity changes (gradients) and the transmission is not constant in these regions, a more careful analysis on the local characteristics of such regions is desired, thus the need for smaller patch sizes.

### 2.4. A Novel Contrast-Guided Approach for the Calculation of Dark Channels and Transmission Maps Using Patches of Varying Sizes

We propose a solution that assigns different patch sizes for different regions in the image based on the level of intensity change observed inside each candidate patch size. The standard deviation σ is chosen as guiding parameter of the proposed approach because its value reflects how strong the contrast is inside a region. Since haze-less images have more contrast [24], the regions where the contrast is found to be high are less affected by haze, and therefore they need less correction (dehazing). Additionally, the regions where coarse textures are present (requiring smaller patch sizes) can also be determined by looking at the standard deviation. This contrast-guided approach solves three problems: radiance oversaturation, undesired dehazing artifacts, and robustness to variable image sizes.

We study σ inside different-sized patches centered at the same pixel of the hazy image (i.e., inverted version of the low-lighting underwater image). If varying the patch size results in an increase of the standard deviation, the variation is undesired. Given that the most commonly used patch sizes observed in the literature range from 3×3 to 15×15 [40] (and also considering that patch sizes bigger than that significantly increase the processing time), seven different options of patch sizes are considered in the contrast-guided approach: 3×3, 5×5, 7×7, 9×9, 11×11, 13×13 and 15×15. Based on the σ value inside each candidate patch size, the *contrast code image* (CCI) is created.

The CCI calculation process is illustrated in Algorithm 1. The seven values of σ for each spatial coordinate (x,y) in the hazy image are calculated and the lowest one is stored in the same position (x,y) of the two-dimensional matrix Sigmas. A second two-dimensional matrix, CCI, stores an integer code *c*(1≤c≤7) referring to the patch size that resulted in the smallest σ. The codes refer, respectively, to patches sized from 3×3 to 15×15. Note that, since bigger patch sizes are preferred in order to strengthen the DCP, all positions in the CCI are initially populated with code 7. Therefore, if for a given region of an image, all patch sizes resulted in the same σ, the 15×15-sized patch would be selected in our proposed approach. 

**Algorithm 1:** Calculation of the contrast code image (CCI) for the proposed approach.

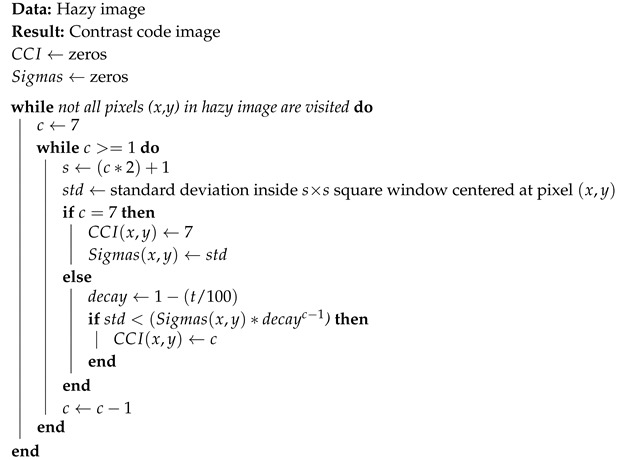



To further stimulate the usage of bigger patch sizes, an additional parameter, tolerance *t*, is introduced. This parameter represents the percentage by which a subsequent σ has to be smaller than the previous one in order to trigger a new code *c* to be stored in the CCI. Tolerance *t* should be carefully chosen; a tolerance of 5% means, for example, that, in order for the algorithm to select a 3×3-sized patch instead of a patch of size 15×15, the smaller patch would have to generate a σ of 73.51% or less than the value of the σ from the bigger patch size. In practice, parameter *t* will increase the number of high-valued patch sizes chosen throughout the image, as exemplified in Section 3.2.1.

The contrast code image works as a guidance tool for the dark channel and transmission map creation. In both these tasks, the size of patch Ω used for each pixel is no longer constant; instead, it is based on the code *c* that the contrast code image possesses in that particular position. In the contrast-guided approach, the size of patch Ω is given by:

(8) $$\Omega_{size}=\left(CCI\left(x,y\right)\times 2\right)+1$$

where CCI(x,y) denotes the integer value of code *c* in this spatial position. Patch Ω is then determined as a square window of dimension size×size. Once the patch sizes for each region are calculated in the CCI, Equations (2) and (3) are now used with patches of appropriate size for each region of the image in the calculation of the dark channel and transmission map. Combining a novel method that considers local contrast information to determine appropriate patch sizes; the knowledge about an ideal transmission map refinement technique and its parameters [33]; and the observation that darkness can be interpreted as haze in inverted images, we developed a robust low-lighting underwater image enhancer. Figure 1 summarizes the entire contrast-guided enhancement process proposed.

## 3. Experimental Results

This section details the experimental validation of the proposed approach. Section 3.1 describes the new low-lighting underwater image dataset, called *OceanDark* [42]. Section 3.2 discusses the performance of the novel contrast-guided approach for image dehazing, which is a critical step for the low-lighting image enhancement framework. Section 3.3 evaluates the performance of our proposed framework on *OceanDark*. Finally, Section 3.4 evaluates the performance of the proposed framework against four state-of-the-art underwater-specific image enhancement methods [18,19,21,22].

### 3.1. The Oceandark Dataset

Multiple datasets composed by underwater images are available online, for example: TURBID [43], which offers hazy underwater images; the samples used in color restoration processes in [19]; the various datasets from the National Oceanic and Atmospheric Administration (NOAA) [44]; and the underwater stereo vision videos from MARIS [45]. However, to the best of our knowledge, no dataset provides samples of both underwater *and* low-lighting scenes simultaneously, although such cases are very frequent, for reasons outlined above. Scenes where the lighting sources are exclusively artificial are of particular interest for this project, as this is the standard setting of most monitored deep-sea underwater sites. The novel dataset proposed, *OceanDark*, responds to this need.

Ocean Networks Canada offers digital platforms for the viewing [46] and downloading [47] of historic and live data from their catalogue of sensors. For the *OceanDark* dataset, 183 image samples of low-lighting underwater images captured using artificial lighting were selected from video footage of ONC’s cameras located in the Northeast Pacific Ocean by downloading the original videos, and then selecting specific frames. Images in the dataset have 1280×720 pixels and portray the following scenarios:

1. Low-lighting underwater images with artificial lighting sources: Sites located deep into the ocean usually contain dark regions that hide valuable information in the images.
2. Images with meaningful structures: To evaluate cases where the proposed enhancement framework is most useful, all samples in the dataset contain large objects, either biological or artificial, that suffer from sub-optimal illumination, e.g., skates, crabs, fish, urchins, scientific apparatus, etc.

**Figure 5:**
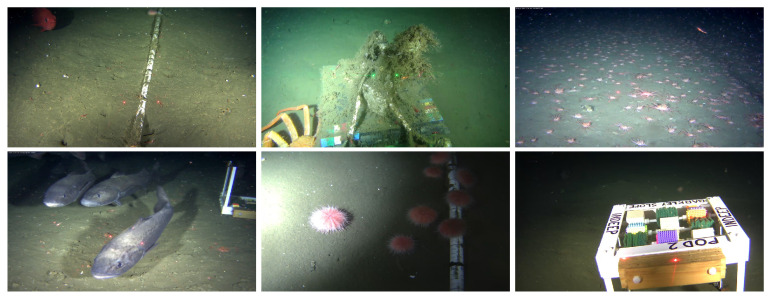
Samples of low-lighting underwater images from the *OceanDark* dataset. All samples in the dataset portray scenes with considerable poorly-lit regions that were captured with the use of artificial lighting.

The primary goal of the dataset is to help evaluate how the proposed framework would perform under real-world low-lighting underwater images. The *OceanDark* dataset does not provide a ground truth reference for the image enhancement framework, given that, for the samples being analyzed, there are no counterpart images with “optimal lighting conditions” available—that is, we are limited to the images captured from the videos as they exist in the archive. Therefore, the evaluation of the proposed framework using *OceanDark* was performed based on other metrics, as detailed in Section 3.2. Figure 5 presents samples from *OceanDark*.

### 3.2. Contrast-Guided Approach Evaluation

This subsection starts by analyzing a low-lighting underwater image to illustrate a scenario where the contrast-guided approach is highly desirable in the dark channel and transmission map calculation steps. Moreover, it presents three experiments that highlight the advantages of using the proposed approach against the standard, single-sized patches [20].

#### 3.2.1. Case Study

**Figure 6:**
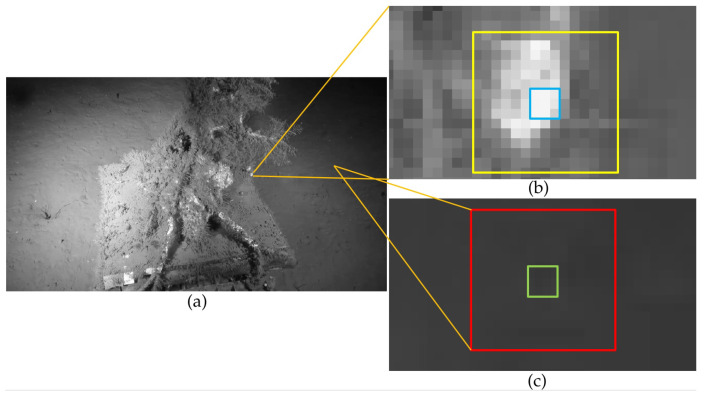
Local standard deviations calculated in regions with homogeneous and non-homogeneous intensities using patches of different sizes (small windows represent patches of size 3×3 while larger ones correspond to patches of size 15×15). Subfigure (**a**) presents a gray-scale version of an *OceanDark* sample. In sub-region (**b**), the blue and yellow windows have σ of 12.70 and 49.55, respectively. This significant difference justifies the use of the smaller patch size. The green and red windows of sub-region (**c**), however, have similar σ of 1.80 and 1.76. In this case, the use of the larger patch is appropriate, given the DCP.

Figure 6 illustrates distinct image regions with varying content that call for variable patch sizes. This gray-scale version of an *OceanDark* sample possesses both homogeneous parts (darker regions of the image), where larger patch sizes are selected in the CCI, and complex textures and edges (closer to its center), where smaller patches are the most suitable (as shown in Table 1). Both smaller or bigger patch sizes (e.g., 3×3 and 15×15) are appropriate to be used in the dark channel and transmission map calculations of homogeneous regions, such as that of Figure 6c. The standard deviation of this region for 3×3- and 15×15-sized patches (green and red windows of Figure 6c) is, respectively, 1.80 and 1.76. Since both patches produced similar results (given the homogeneous content of this region), the bigger patch usage is preferred, as discussed in Section 2.4.

Conversely, the region highlighted in Figure 6b has significant intensity gradients that call for a more meticulous analysis of the lighting profile (transmission map), since the intensity from this region is not homogeneous. The 15×15-sized patch (white window of Figure 6b) interprets a bigger, heterogeneous region as being a single unit. The varying lighting profile inside this bigger patch is reflected by its high standard deviation, 49.55. On the other hand, using a smaller patch size of 3×3 (blue window of Figure 6b) leads to a significantly smaller standard deviation of 12.70.

Clearly, there is no single selection of patch size that would correctly fit the whole image. The CCI calculated with t=0 in Figure 6a has 704,622 (76.4%) codes *c* for 3×3-sized patches, revealing that for 23.6% of the image’s pixels, this smaller patch was not the best option. If parameter *t* is set to 7%, the number of non-3×3 patches chosen in the CCI of Figure 6 grows to 39.79%. This increase in patch size selection strengthens the DCP, leading to more accurate dehazing results. Excessively big patch sizes create, however, unwanted dehazing artifacts (as discussed in Section 2.3. Table 1 summarizes the findings of this experiment.

#### 3.2.2. Comparison between the Usage of Dynamic and Static Patch Sizes

Each of the advantages brought by the novel contrast-guide method when compared to the use of single-sized patches are discussed in the following experiments.

**Figure 7:**
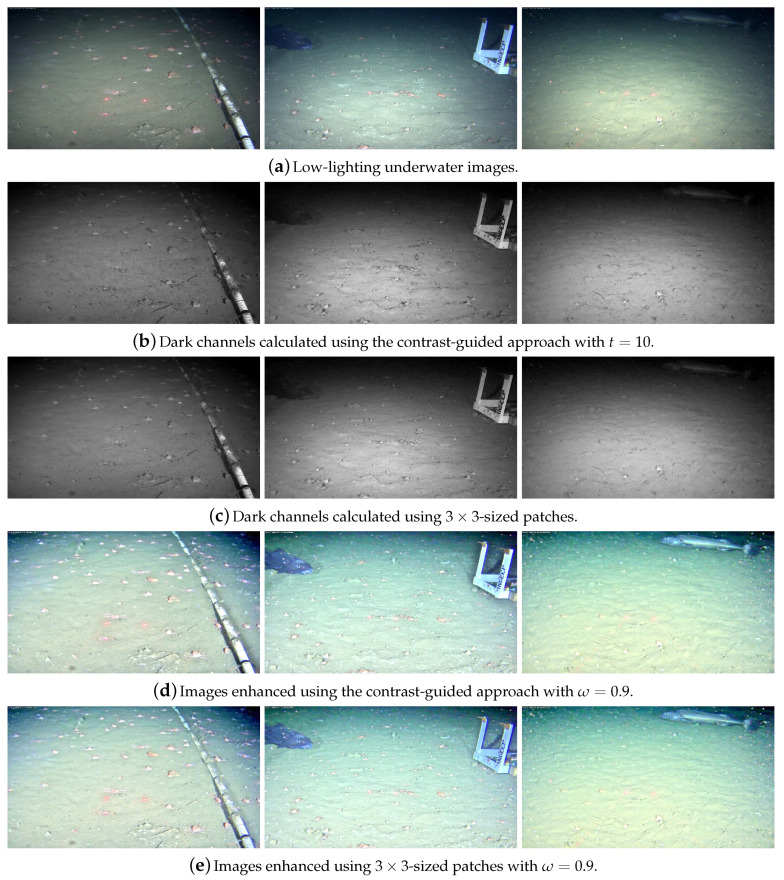
Comparison between dark channels and enhanced images calculated using single-sized patches of 3×3 and the contrast-guided approach. The proposed method produces darker DC (**b**) when compared to those obtained with 3×3-sized patches (**c**), which is preferred based on the DCP. The outputs of the proposed method (**d**) have higher contrast—a characteristic desired in dehazed images [24,51].

**Radiance oversaturation and loss of detail**. Figure 7 compares images dehazed using the contrast-guided approach and small, fixed patches of size 3×3. From the left to right columns of Figure 7, the OceanDark samples are referred to as “OceanDark 1” through “OceanDark 3”. The metrics chosen to compare the use of single-sized patches and the proposed approach in terms of saturation of colors and loss of detail (quality) are the mean intensity of the dark channels created, the number of Speeded Up Robust Features (SURF) points [48] observed after the enhancement processes and, given the lack of ground truth data, two no-reference scores: BLIINDS-II [49] and Global Contrast Factor (GCF) [50]. The parameters used in this experiment are ω=0.9 and tolerance t=10. Table 2 shows that the mean pixel intensity of the dark channels created using the contrast-guided approach (Figure 7b) is lower, because darker pixels (lower intensity) are selected in their calculation. The dark channels created using the contrast-guided approach better highlight the layout of the low-lighting images, when compared to the ones obtained with static patch sizes.

Since the low-lighting scenes that were analyzed contain both homogeneous and non-homogeneous regions, their CCI contain a significant number of codes *c* representing not only 3×3 patches, but also the six other possible sizes (see Table 2). In particular, the CCI of the OceanDarks 1–3 had 48.55%, 36.2% and 32.48% of *c* codes *not* representing 3×3-sized patches, respectively. The larger patches are chosen in regions where the transmission is approximately constant, as discussed in Section 3.2.1.

To quantify the improvement in the dark channel calculation, two reference values are considered: the mean pixel intensity of dark channels created using exclusively 3×3 and 15×15 patch sizes. The first should offer the highest mean intensity to be considered, here called “higher reference”, while the latter yields the lowest mean intensity, or “lower reference”. Given that the contrast-guided approach calls for the use of patch sizes valued between these two options, its dark channel’s mean intensity will necessarily sit between the two measures. Therefore, an “improvement” score of 0% is attributed to dark channels where the mean intensity matches the higher reference (which is not desirable), while 100% would represent mean intensities that match the lower reference. Table 2 shows a significant improvement in the dark channels of all *OceanDark* samples when using the contrast-guided approach.

The number of feature points measured after the enhancement process is also analyzed in Table 2. This is a commonly employed metric to evaluate the quality of dehazed images [40,52]. Feature points provide a strong indicator of the usefulness of an image for multiple higher-level computer vision methods, such as motion tracking, object detection and shape detection. The number of feature points is calculated using the SURF [48] method. As detailed in Table 2, all samples dehazed using the contrast-guided method present more SURF features than those dehazed using fixed-size patches.

Haze-less images have greater contrast than those that are hazy [24,53] (that is also true for low-lighting/enhanced underwater images). A qualitative analysis of the results shows that the images enhanced using only 3×3-sized patches have less contrast (Figure 7d) than those generated using the contrast-guided approach (Figure 7e). That happens because the haze is overestimated by a less accurate DCP when using the 3×3-sized patches (as detailed in Section 2.3). A recent work [54] used the no-reference, discrete cosine transform-based BLIINDS-II [49] algorithm to evaluate dehazing results of multiple methods. We use that same metric to compare the results of this experiment, and, in addition to that, we calculate the contrast-based GCF score [50] to compare the contrast level of the enhanced underwater images. Higher scores in BLIINDS-II and GCF represent, respectively, images of higher quality and images with higher contrast. Table 2 shows that both scores are higher in all the samples enhanced using the proposed approach.

The incorrect estimation of haze denseness caused by a less accurate DCP is also manifested in the form of oversaturated scene radiance (i.e., pixel intensity) in the enhanced images, as noted by He et al. [20]. Qualitatively, the images enhanced with $\Omega_{3\times 3}$ (Figure 7e) become excessively bright, while those in Figure 7d present a scene radiance that more closely matches the original images. In experiments with aerial images, He et al. [20] illustrated the oversaturation issue by comparing images dehazed using $\Omega_{3\times 3}$ and $\Omega_{30\times 30}$ patches, while Cheng et al. [41] used $\Omega_{45\times 45}$ patches to avoid this phenomenon. Our method also empirically shows a reduction in oversaturation of radiance, this time for low-lighting underwater images. We chose $\Omega_{15\times 15}$ as the largest patch size in our method to prevent higher processing times and to validate our approach. However, simply increasing the number of larger patch sizes considered by our proposed method would mitigate even more the oversaturation effects.

**Figure 8:**
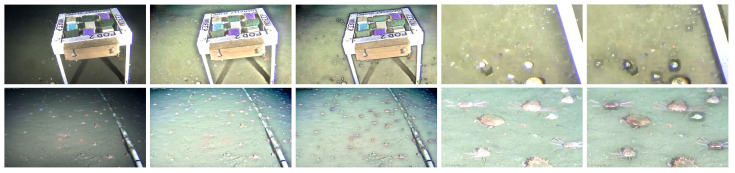
Enhanced *OceanDark* samples using 15×15-sized patches and the contrast-guided approach. Columns from left to right: low-lighting samples, images enhanced using the contrast guided approach and 15×15-sized patches (respectively), zoomed-in regions from the images obtained with the contrast-guided approach and 15×15-sized patches (respectively). The halos observed when using single-sized patches are considerably reduced in the output of the proposed method.

**Dehazing artifacts (halos)**. A second problem addressed by the proposed approach is the presence of undesired artifacts resulting from dehazing processes that use larger patch sizes (e.g., patches of 15×15 used in the original work of He et al. [20]). The contrast-guided approach proposed selects different patch sizes for distinct regions of the image, greatly attenuating such artifacts. Figure 8 compares the results of the enhancement process when using the contrast-guided approach and 15×15-sized patches.

This experiment’s parameters are ω=0.9 and tolerance t=0. The images in the third column of Figure 8, which were obtained using patches of 15×15, illustrate enhancement results where multiple halos are noticed around strong intensity gradients (e.g., rocks, shells and scientific apparatus). Such halos can mislead methods that rely on the detection of edges and feature points. The proposed method considerably reduces these undesired enhancement artifacts (second column Figure 8). Columns 4 and 5 of Figure 8 highlight, respectively, regions of the enhanced images were halos are considerably lower when comparing the contrast-guided and the fixed patch size approach. Since bigger patches create new (invalid) edges and features in the enhanced images, metrics that consider those parameters are not applicable in this experiment.

**Figure 9:**
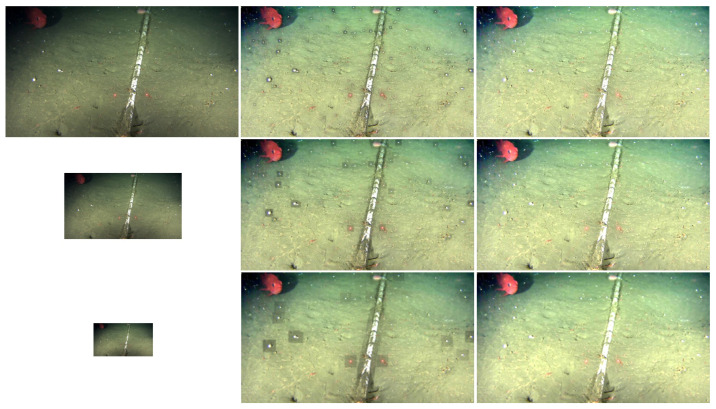
Different scales (100%, 50% and 25% in Rows 1–3, respectively) of the same low-lighting underwater sample from *OceanDark* enhanced using the contrast-guided approach with t=5 and patches of $\Omega_{15\times 15}$. Left column: original images in different scales. Center column: Enhancement results using single-sized patches of $\Omega_{15\times 15}$. Right column: Enhancement results using the contrast-guided approach. The proposed method does not create noticeable halos in any of the image scales because their distinct CCI will indicate the best sizes for patches Ω to be used in each of them. The fixed-sized patches approach created increasingly large rectangular halos around regions with significant intensity gradients, introducing false edges and features.

**Scale-invariance**. A major contribution of the contrast-guided approach proposed is that it can be used for images of vastly different sizes. While patches of fixed sizes (e.g., $\Omega_{3\times 3}$ and $\Omega_{15\times 15}$) might be appropriate for an image of certain dimensions, this same choice of patch size might prove to be inadequate for the same image under different scales, as illustrated in Figure 9. To observe the effects of using the same patch size for different image dimensions, the original image (1280×720 pixels) was reduced by 50%, down to 640×360 pixels, and then once again reduced by 50%, to a final size of 320×180 pixels. These different scales are illustrated on the leftmost column of Figure 9. The parameters used in this experiment are: static patch size of 15×15, ω=0.9 and t=5.

To facilitate visual comparison, the enhancement outputs for smaller scales were resized in the central and right columns of Figure 9. Note that difference in halo sizes created around small regions with strong gradients (e.g., rocks) for the enhanced outputs of the original image scale are minor using both approaches. The halos grow significantly for the reduced scales when using the fixed-size patch, since these $\Omega_{15\times 15}$ patches encompass regions of increasingly more heterogeneous transmission (pixel intensities), weakening the assumption that a single value for a large patch could correctly represent them. On the other hand, the contrast-guided approach adjusts to these new dimensions, and selects appropriate patch sizes for each region of the image, reducing the halos created on the outputs. This powerful characteristic of the contrast-guided approach allows it to be used with images of any size, regardless of their content.

Table 3 shows the distribution of patch sizes used in the contrast-guided approach for the experiment depicted in Figure 9. A significant number of patches with size larger than 3×3 (approximately 35%) is used in all scales, reflecting the method’s ability to select appropriate patch sizes based on local contrast information. Note that the number of smaller patch sizes chosen for smaller scales proportionally increases. This expected behavior happens because, as the scale lowers, smaller patch sizes are capable of generating the smallest standard deviations.

### 3.3. Enhancement Framework Evaluation with Low-Lighting Underwater Images

**Figure 10:**
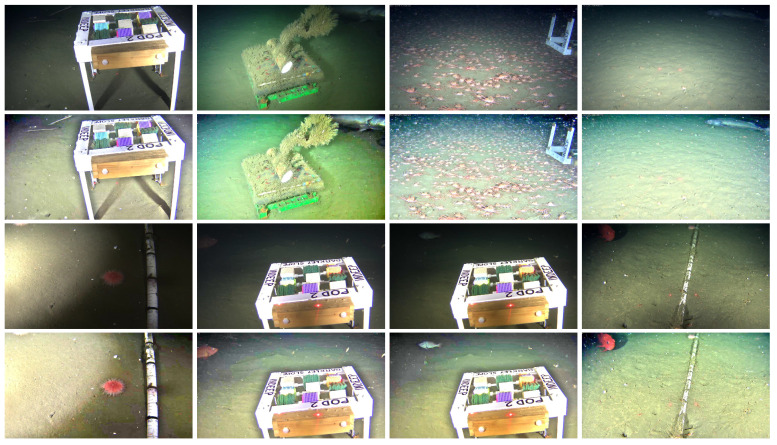
Enhancement of low-lighting underwater images from *OceanDark* using the proposed method. Rows 1 and 3: Low-lighting underwater images. Rows 2 and 4: Enhanced images. The enhanced images have more contrast and better lighting, as well as highlight important structures (i.e., animals, rocks, and man-made scientific apparatus) that can be used for further analysis of the visual data.

This subsection presents and discusses examples of low-lighting underwater images from the *OceanDark* dataset that are enhanced using the CCI as part of the proposed framework. Three metrics are used to quantitatively evaluate the results. Figure 10 illustrates eight examples of low-lighting underwater images from the *OceanDark* dataset whose enhancement would greatly benefit a subsequent analysis by a human annotator or automated analysis.

The parameters used in the enhancement presented in Figure 10 are: ω=0.9, tolerance t=3, guided filter subsampling ratio s=4, square window radius r=28 and smoothing degree ϵ=0.45. A qualitative analysis of Figure 10 shows that the main goal of the enhancement framework is achieved: objects that were initially difficult to notice in Rows 1 and 3, such as skates, crabs, fish, urchins, rocks, and details of the human-made scientific apparatus, become apparent in the enhanced versions of the samples shown in Rows 2 and 4. The enhanced images can aid both human and automated analysis by offering better visibility: the originally dark regions are illuminated, the overall contrast of the images is increased, and the pixel intensity gradients are stronger. The framework preserves the edges of the original images thanks to the use of the CCI, which selects the best patch size for each pixel-centered window of the input image, and the fast guided filter [34], an edge-preserving image filter.

The enhancement framework is applied to all images of the *OceanDark* dataset (using the same parameters as those for the results presented in Figure 10) so the relationship between original and enhanced images can be analyzed. Since there are no ground truth references for the data from *OceanDark*, full-reference methods that compare similarity between images are not applicable. Four metrics commonly used to assess dehazing frameworks [24,40,55] were employed to perform a quantitative analysis of the results.

1. SURF features [48]: As discussed in Section 3.2.2, the increase in number for these features indicate a boost in usefulness of the underwater images.
2. *r*-score [56]: This score compares the visibility level of both original and enhanced images. Thus, an increase in *r*-score represents a successful contrast-enhancing procedure.
3. *e*-score [56]: The increase in number of visible edges between the original and enhanced images is used to calculate this score. Edges, similar to SURF features, can be used for detection, tracking, among others. The Canny edge detector [57] was used to detect the edges.
4. Fog Aware Density Evaluator (FADE) score [58]: This is a no-reference score based on the perceptual fog density observed in an image. As discussed in Section 2.1, the low-lighting regions of an image are considered as haze in its inverted version. Therefore, the images are inverted before this score is measured, allowing for the study of their fog (or haze) levels. Smaller scores represent less fog (for our case study, darkness), which is desired.

The same parameters used for Figure 10 were used in this experiment. The strong and weak thresholds used with the Canny edge detector were, respectively, 0.2 and 0.05. Table 4 shows a significant increase in the number of SURF features and *e*-score (106.84% and 28%, respectively), which indicates that more meaningful visual structures, originally hidden by dark regions, became apparent after the enhancement process. Caution should be used when considering new edges and features created, given that some of them might represent noise added to the image. As expected, the visibility enhancement indicator *r*-score presented an increment of 175%, attesting to the contrast-guided method’s ability to recover images with better lighting. Finally, the significant decrease of 75.92% measured for the FADE score indicates that the dark regions of the original images were greatly reduced, revealing data initially hidden in the *OceanDark* samples.

### 3.4. Comparison with State-of-the-Art Underwater-Specific Enhancement Frameworks

**Figure 11:**
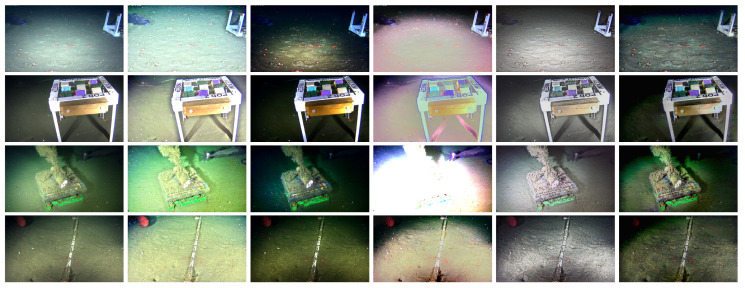
Comparison between underwater-specific image enhancement frameworks applied to samples 104 (first row), 11 (second row), 75 (third row) and 181 (fourth row) of *OceanDark*. First column: Original low-lighting images. Columns 2–6: Output from the proposed approach, Drews et al. [18], Berman et al. [19], Fu et al. [22] and Cho and Kim [21], respectively. The parameters used with the proposed method are t=3 and ω=0.9. The parameters used with the other methods are the authors’ default values provided in the official implementations.

Four state-of-the-art underwater-specific enhancement frameworks [18,19,21,22] were compared with the proposed method to further evaluate its performance. We chose Drews et al. [18] method, which also modified the original DCP (by disregarding the influence of the red color channel) to work with underwater images. The other three works (Berman et al. [19], Fu et al. [22] and Cho and Kim [21]), discussed in Section 1.1, were selected to allow for a comparison with enhancement methods that are not based on the Dark Channel prior. The implementation code for all methods were made publicly available by their respective authors. Figure 11 shows typical enhancement results of the proposed method and the compared methods on four *OceanDark* images (from top to bottom rows: Samples 104, 11, 75 and 181, respectively). Five previously discussed metrics (GCF [50], *e*- and *r*-score [56], FADE [58] and SURF [48]) were measured on the output of all methods over all samples composing *OceanDark*. The aggregated values (mean and standard deviation) of such results are presented on Table 5.

Drew’s method [18] enhanced the samples’ contrast, enabling for a better distinction of the edges that were already visible in the original images (high GCF and *e*-score). However, low-lighting regions became even darker, further occluding important details of the image, as highlighted by its high perceived darkness (a FADE score higher than the original samples) and low visibility (*r*-score). The output obtained with the framework of Berman et al. [19] introduced unnatural colors on the *OceanDark* samples, a phenomenon that happens because of the distinct water types that this method chooses from for each individual sample. Berman’s high visibility score (*r*-score) is credited to samples where the selected water type generated an oversaturated output (e.g., Berman’s output for Sample 75 in the third row of Figure 11). The Retinex-based model of Fu et al. [22] highlighted regions with intensity gradients (e.g., seabed rocks from Sample 181), resulting in a high SURF features count. However, it provided only marginally improved visibility for low-lighting regions (FADE score similar to the original samples). Its low-contrast output also led to low *e*-scores. Similarl to the results of Drews et al. [18], Cho and Kim method’s [21] high-contrast output enhanced edges, in particular in regions with originally good visibility (high CGF and *e*-score). On the other hand, low-lighting regions also become darker, virtually hiding important visual information (such as the ray fish from Sample 11), which lowered its *r*-score while increasing the FADE score. Our proposed approach (whose performance is thoroughly discussed in Section 3.3) drastically reduced the perceived darkness (75.92%) and increased the *e*-score by 28%. The contrast-guided approach also increased by 175% the *r*-score, enhancing the visibility in regions that were initially dark. As an example, the sable fish and ray fish from Samples 101 and 11 (respectively) became clearly visible after the image enhancement. The halos created around intensity edges are minimal thanks to the contrast-guided enhancement approach.

The poor results in the visibility-related metrics (FADE and *r*-score) from most of the methods evaluated is due to the fact that none of them were developed with low-lighting scenarios in mind. While these methods offer elegant and well-suited solutions for underwater images with regular lighting levels, our proposed method focuses on those that are sub-optimally illuminated. The high performance of our method with respect to low-lighting scenarios, which are not handled well by the considered related works, highlights the usefulness of our approach. One should note that low-lighting scenarios are quite common in deep-sea imagery.

## 4. Conclusions

This article presents a novel low-lighting underwater image enhancement framework that is based on local contrast information. The proposed framework performs a novel dehazing step that was designed to address important limitations of other single-image dehazing methods: radiance oversaturation, loss of detail, lack of scale-invariance and creation of halos.

To design and evaluate the enhancement framework, a low-lighting underwater image dataset called *OceanDark* [42] has been created and made available. Experimental results show that the contrast-guided enhancement process outperforms methods that employ static patch sizes. A qualitative analysis of the enhanced low-lighting underwater images reveals that the proposed method can significantly increase the meaningful information in the images by enhancing their lighting, contrast, and ultimately the image quality (see Section 3.2.2), allowing for better interpretation of such data. Quantitatively, the number of SURF features [48], *r*-score and *e*-score [56] increased when comparing original and enhanced images by, respectively, 106.84%, 175% and 28%. Moreover, the perceived darkness (FADE score [58]) decreased, on average, 75.92% in the enhanced images. A comparison with four state-of-the-art underwater-specific image enhancement frameworks showed that the proposed method is very well suited for low-lighting scenarios.

The extent to which the proposed method can mitigate the patch size-related problems, in particular the radiance oversaturation, is currently limited by the number of sizes (7) considered in the CCI calculation. The method can be easily expanded to account for other patch sizes (i.e., larger than 15×15), as explored in other works [20,41]. This choice would slow the framework’s processing time, however better radiance oversaturation and halo prevention would ensue.

The following opportunities for future work and improvements to the method have been identified. Firstly, the proposed method does not consider chromatic information of the inputs, which may result in color fidelity problems in the output images. Future work will focus on preserving chromatic information during the image enhancement process. Secondly, the atmospheric lighting *A* is represented as a single value in each color channel. This simplification will be corrected by considering non-homogeneous lighting sources (i.e., multiple values of *A* for distinct regions of the image) in later iterations of the proposed method. Thirdly, improvements to the method addressing oversaturation of radiance could be made by considering larger patch sizes and applying quantitative metrics for evaluation.

## Figures and Tables

**Table 1:** Analysis of the standard deviation in the gray-scale version of an *OceanDark* sample.

	σ for $\Omega_{3\times 3}$	σ for $\Omega_{15\times 15}$	3×3-Sized Patches	Non-3×3-Sized Patches
Sub-region of Figure 6b	12.70	49.55	N/A	N/A
Sub-region of Figure 6c	1.80	1.76	N/A	N/A
Whole Figure 6a with t=0	N/A	N/A	704,622 (76.4%)	216,378 (23.6%)
Whole Figure 6a with t=7	N/A	N/A	554,534 (60.21%)	366,466 (39.79%)

**Table 2:** Comparison between dark channels, feature points, quality and contrast of enhanced images when using the contrast-guided approach and 3×3-sized patches. The proposed approached achieved better results (lower mean pixel intensities, higher CGF scores and higher number of SURF features) in all the metrics used.

	$\Omega_{3\times 3}$ Patches Used	$\Omega_{15\times 15}$ Patches Used	DC ${}^{1}$ Mean Pixel Intensity	DC ${}^{1}$ References ($\Omega_{3\times 3}$/$\Omega_{15\times 15}$)	DC ${}^{1}$ Improvement	GCF Score	BLIINDS-II Score	SURF Features
OceanDark 1 with CGA ${}^{2}$	474,201 (51.45%)	265,663 (28.82%)	**77.89**	80.80/71.28	**29.01**%	**0.1985**	**77.5**	**582**
OceanDark 1 with SA ${}^{3}$	921,600 (100%)	0	80.80	80.80/71.28	N/A	0.1792	76.5	560
OceanDark 2 with CGA ${}^{2}$	587,982 (63.80%)	177,824 (19.29%)	**95.89**	98.11/87.04	**20.01%**	**0.1928**	**73**	**530**
OceanDark 2 with SA ${}^{3}$	921,600 (100%)	0	98.11	98.11/87.04	N/A	0.1757	71.5	495
OceanDark 3 with CGA ${}^{2}$	622,323 (67.52%)	154,447 (16.76%)	**97.07**	98.55/90.86	**19.22%**	**0.1794**	**72**	**185**
OceanDark 3 with SA ${}^{3}$	921,600 (100%)	0	98.55	98.55/90.86	N/A	0.1738	71	172

^1^ Dark channel [20]. ^2^ Dark channel, transmission map and enhanced image obtained using the contrast-guided approach. ^3^ Dark channel, transmission map and enhanced image obtained using $\Omega_{3\times 3}$ patches.

**Table 3:** Distribution of patch sizes used in the enhancement of an *OceanDark* sample at different scales an t=5.

	$\Omega_{3\times 3}$	$\Omega_{5\times 5}$	$\Omega_{7\times 7}$	$\Omega_{9\times 9}$	$\Omega_{11\times 11}$	$\Omega_{13\times 13}$	$\Omega_{15\times 15}$
100%	65.45%	9.56%	4.03%	2.48%	2.11%	2.18%	14.16%
50%	65.09%	8.34%	4.49%	3.08%	2.68%	2.64%	13.63%
25%	65.18%	10.51%	5.09%	3.28%	2.82%	2.60%	10.48%

**Table 4:** Quantitative analysis of the contrast-guided enhancement method. The results shown reflect the mean of the measurements from all 183 samples of *OceanDark*.

	Increase in SURF Features	Increase in *e*-Score	Increase in *r*-Score	Decrease in FADE Score (Darkness)
Enhanced images	106.84%	28%	175%	75.92%

**Table 5:** Mean and standard deviation of the five metrics used to compare the proposed method and four state-of-the-art underwater-specific image enhancement methods over all samples of *OceanDark*.

	Original	Proposed	Drews [18]	Berman [19]	Fu [22]	Cho [21]
GCF [50]	3.28±0.62	3.41±0.71	4.70±0.89	3.84±1.07	3.28±0.57	4.11±0.84
*e*-score [56]	N/A	0.28±0.32	1.06±0.83	0.25±0.5	0.09±0.39	0.89±0.54
*r*-score [56]	N/A	2.75±0.76	1.29±0.31	2.91±1.96	1.72±0.25	1.72±0.09
FADE [58]	1.91±0.79	0.46±0.18	2.08±0.90	1.15±0.40	1.75±0.25	1.81±0.52
SURF [48]	340.97±293	705.28±470	589.45±324	425.32±317	865.02±478	751.97±428
